# Inflammatory Arthritis Uncovered Through Imaging of Osteonecrosis: A Case Series

**DOI:** 10.7759/cureus.102491

**Published:** 2026-01-28

**Authors:** Divya Pabbisetti, Anantaram Gudipati, Amber Papalkar, Sandeep Ponnaganti, Kiran Kumar Sailagundla

**Affiliations:** 1 Department of Radiology, Krishna Institute of Medical Sciences, Hyderabad, IND; 2 Department of Radiology, Sacred Heart Hospital, Pensacola, USA

**Keywords:** inflammatory arthritis, kienböck's disease, magnetic resonance imaging, mueller-weiss syndrome, osteonecrosis

## Abstract

Osteonecrosis (ON) is typically viewed as an isolated condition stemming from vascular compromise. We present a three-case series demonstrating that MRI, performed to confirm the diagnosis of ON, incidentally revealed features consistent with underlying, previously undiagnosed inflammatory arthritis (IA). Even in the absence of typical clinical features, the presence of imaging findings such as synovitis, tenosynovitis, and erosions should prompt a thorough clinical and serological evaluation for coexisting IA. An interesting point to note is that none of the three patients reported a history of steroid use. This manuscript aims to highlight a possible pathophysiological link between IA and ON.

## Introduction

Osteonecrosis (ON) is the death of bone tissue resulting from impaired blood supply [1] and is typically identified using imaging modalities such as radiographs and MRI. In some cases, imaging may also reveal features suggestive of inflammatory arthritis, including synovitis, bone marrow edema (BME), and erosions, prompting reconsideration of the underlying diagnosis.

Here, we present two cases of Kienboch’s disease (osteonecrosis of the lunate bone) and one case of Müller-Weiss syndrome (adult-onset osteonecrosis of the navicular bone). In all three patients, plain radiographs initially demonstrated osteonecrosis, and subsequent MRI confirmed the diagnosis while also revealing features consistent with inflammatory arthritis. Laboratory investigations showed elevated inflammatory markers supporting this diagnosis. Notably, none of the patients had a prior diagnosis of inflammatory arthritis or a history of steroid use. This case series explores the potential relationship between osteonecrosis and inflammatory arthritis.

## Case presentation

Case 1

**Figure 1:**
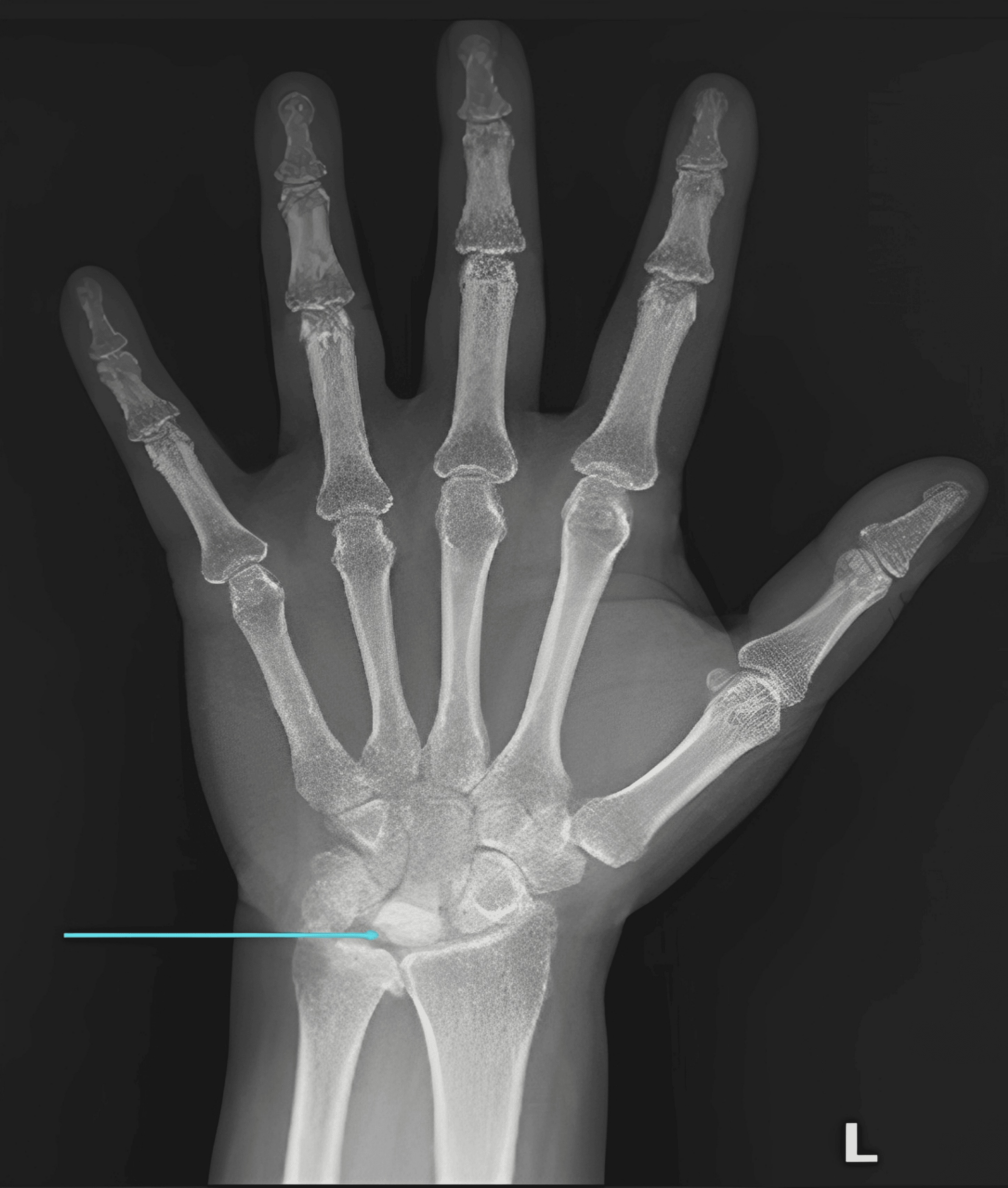
AP radiograph of the left hand showing a partially collapsed and sclerotic lunate (arrow), suggesting Kienbock’s disease

A 51-year-old female presented with a six-month history of left wrist pain and restricted movement. She reported temporary relief of pain with nonsteroidal anti-inflammatory drugs. She was initially evaluated with a radiograph, which showed a partially collapsed and sclerotic lunate, suggesting the diagnosis of Kienbock’s disease (Figure 1).

**Figure 2:**
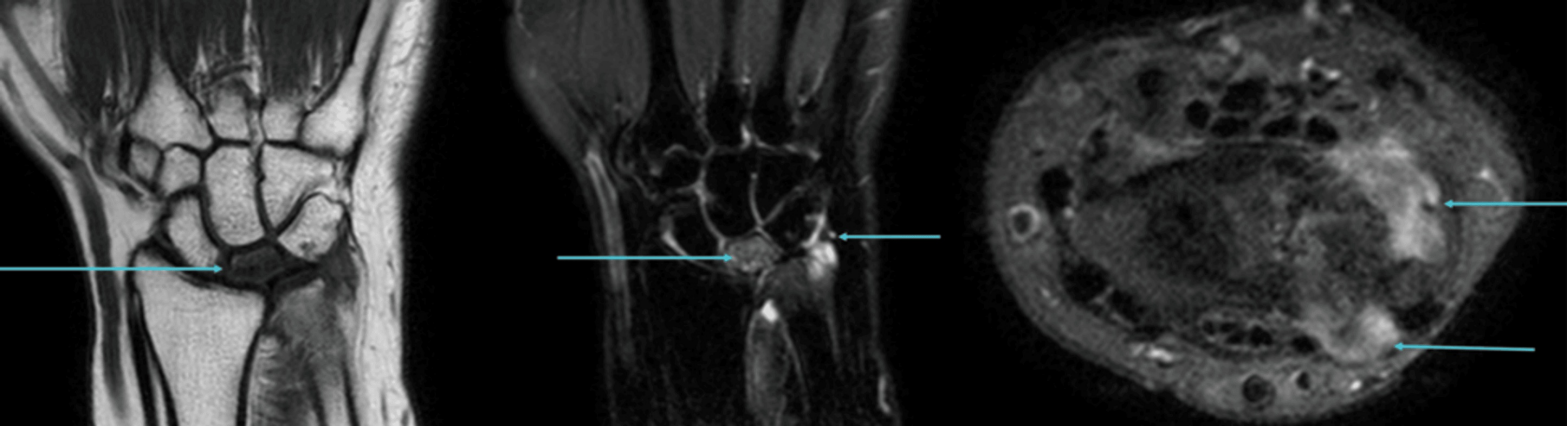
T1 coronal, PD-FS coronal, and axial images (left to right) showing partial collapse of the lunate with diffuse T1 hypointense signal and a chronic coronal fracture line (arrows, left and middle images) Synovitis is present in the distal radioulnar joint, pre-styloid recess, and piso-triquetral joint (arrows, middle and right images). PD-FS, proton density fat-saturated

For confirmation, an MRI was performed. MRI revealed partial collapse of the lunate with diffuse T1 hypointense signal and a chronic coronal fracture line, consistent with Kienbock’s disease. Synovitis was noted involving the distal radioulnar joint, pre-styloid recess, and piso-triquetral joint (Figure 2).

**Figure 3:**
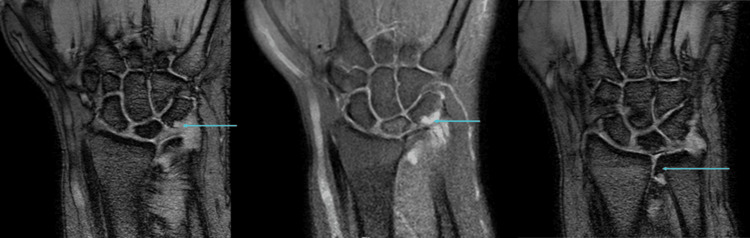
GRE coronal, PD-FS coronal, and GRE coronal images (left to right) showing a few erosions in the triquetrum (arrows, left and middle images) Small osteophytes are seen arising from the distal radioulnar joint (arrow, right image). GRE, gradient-recalled echo; PD-FS, proton density fat-saturated

A few erosions were identified in the triquetrum, and small osteophytes were seen arising from the distal radioulnar joint (Figure 3).

Kienbock’s disease with coexisting IA of the wrist and degenerative changes in the distal radioulnar joint. Laboratory parameters are summarized in Table 1.

**Table 1:** Laboratory parameters of Case 1 ESR, erythrocyte sedimentation rate

Parameter	Value	Normal range
ESR	45 mm/hr	0-20 mm/hr
CRP	1.47 mg/dL	<0.5 mg/dL

The patient received an intramuscular injection of Depo-Medrol 80 mg, following which her pain score decreased from 8/10 to 2/10.

Case 2

**Figure 4:**
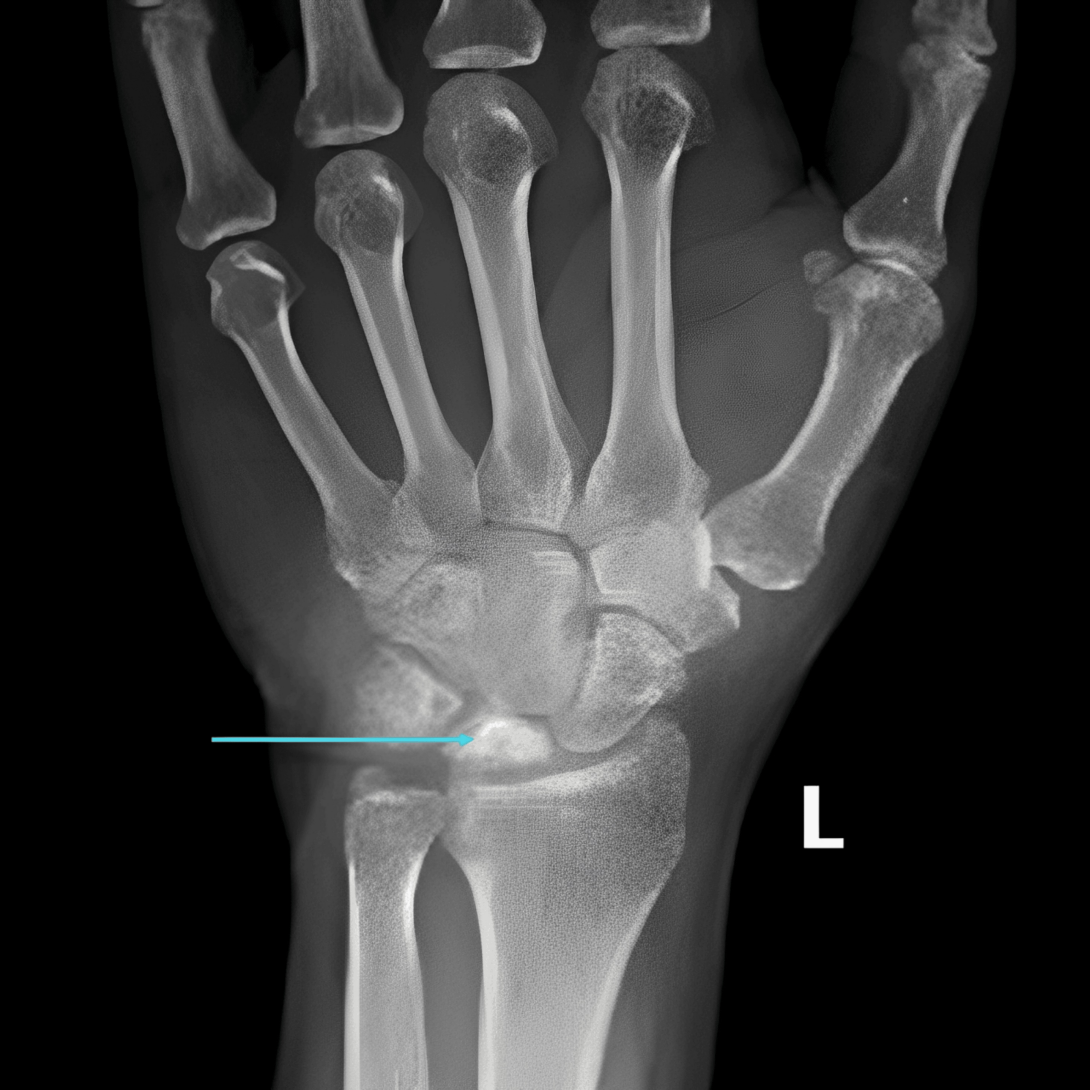
AP radiograph of the left hand showing a partially collapsed and sclerotic lunate (arrow), suggesting Kienbock’s disease

A 46-year-old male presented with a three-month history of left wrist pain and restricted movement. He was initially evaluated with a radiograph, which showed a partially collapsed and sclerotic lunate, suggesting the diagnosis of Kienbock’s disease (Figure 4).

**Figure 5:**
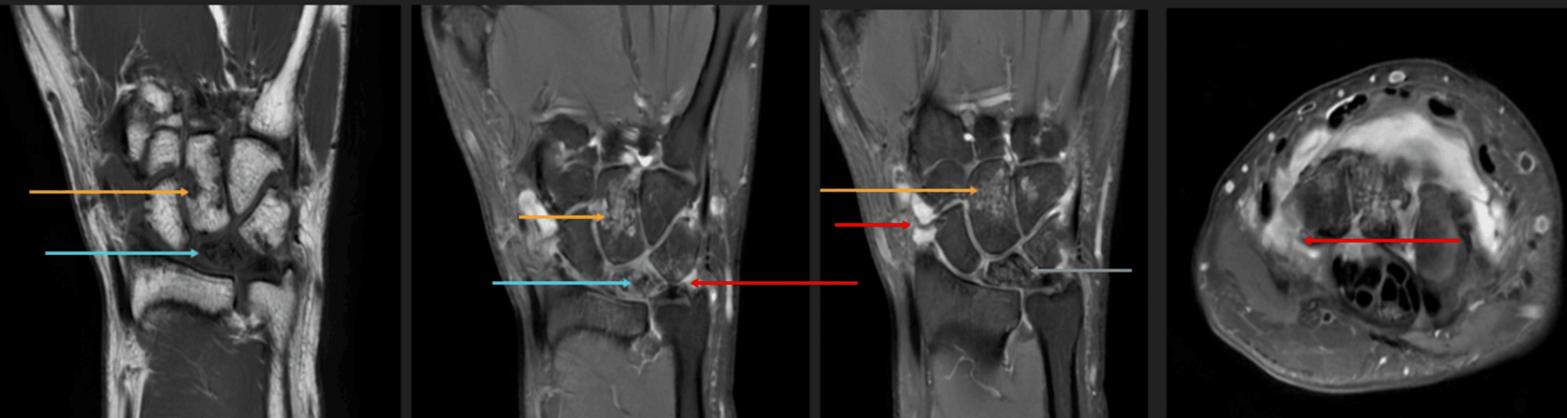
T1 coronal, PD-FS coronal, and axial images (left to right) showing partial collapse of the lunate with irregular fragmentation, diffuse sclerosis, and ill-defined marrow edema (blue arrows) consistent with Kienbock’s disease Synovial thickening and effusion are present in the wrist, intercarpal, and carpometacarpal joints and pre-styloid recess (red arrows). Multiple erosions with associated marrow edema are seen in the triquetrum, capitate, and hamate bones (orange arrows). PD-FS, proton density fat-saturated

**Figure 6:**
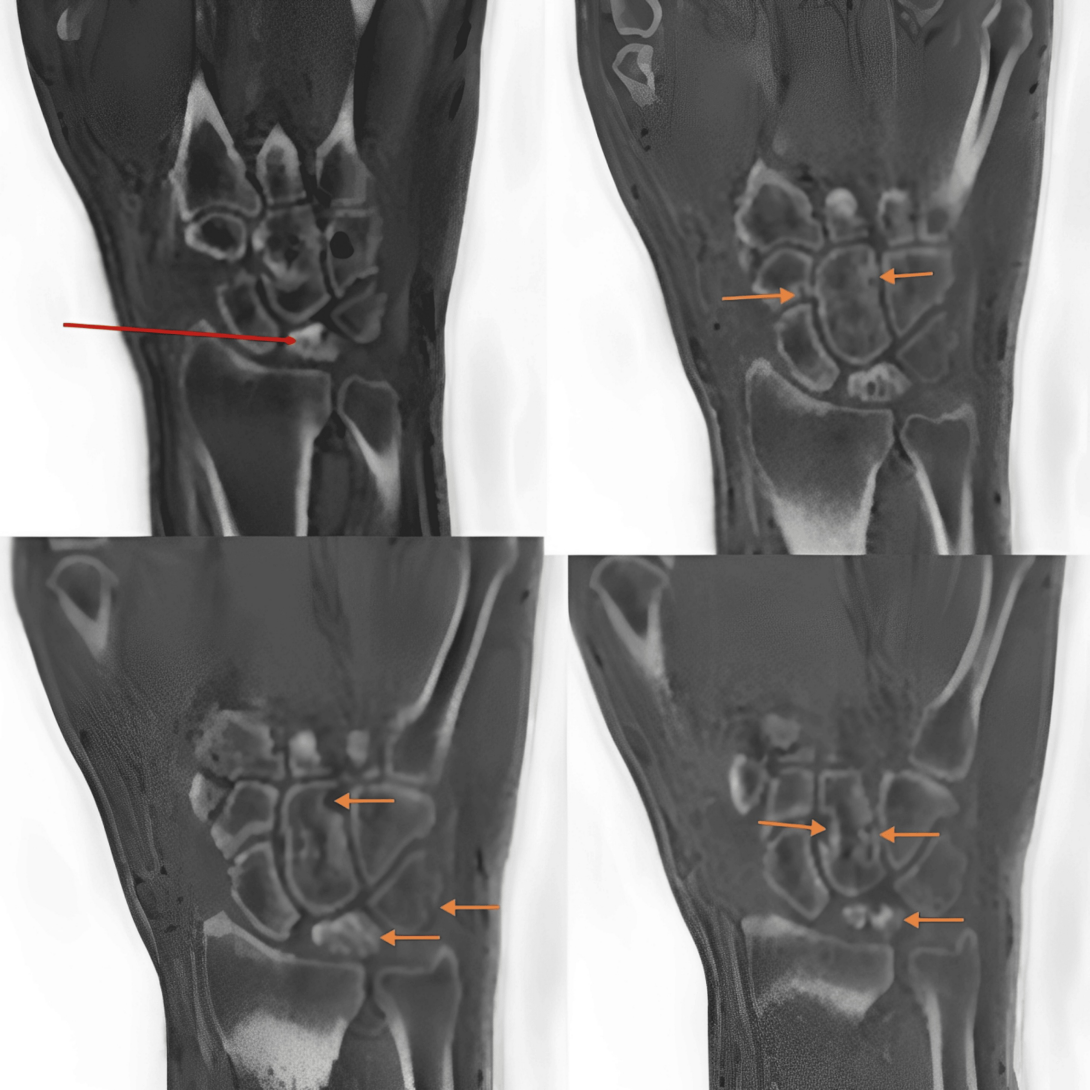
ZTE coronal images showing partial collapse of the lunate with irregular fragmentation and diffuse sclerosis (red and orange arrows, top left, bottom left, and right images) consistent with Kienbock’s disease Multiple erosions are seen in the triquetrum, capitate, and hamate bones (remaining orange arrows). ZTE, zero echo time

For confirmation, an MRI was performed. MRI revealed partial collapse of the lunate with irregular fragmentation, diffuse sclerosis, and ill-defined marrow edema, consistent with Kienbock’s disease. Synovial thickening and effusion were observed in the wrist, intercarpal and carpometacarpal joints, and pre-styloid recess. Multiple erosions with associated marrow edema were noted in the triquetrum, capitate, and hamate bones (Figure 5, Figure 6).

Kienbock’s disease with coexisting IA of the wrist. Laboratory parameters are summarized in Table 2.

**Table 2:** Laboratory parameters of Case 2 ESR, erythrocyte sedimentation rate; RA factor, rheumatoid factor

Parameter	Value	Normal range
ESR	38 mm/hr	0-20 mm/hr
RA factor	125 IU/ml	<14 IU/ml

The patient is being treated for seropositive erosive rheumatoid arthritis (RA).

Case 3

**Figure 7:**
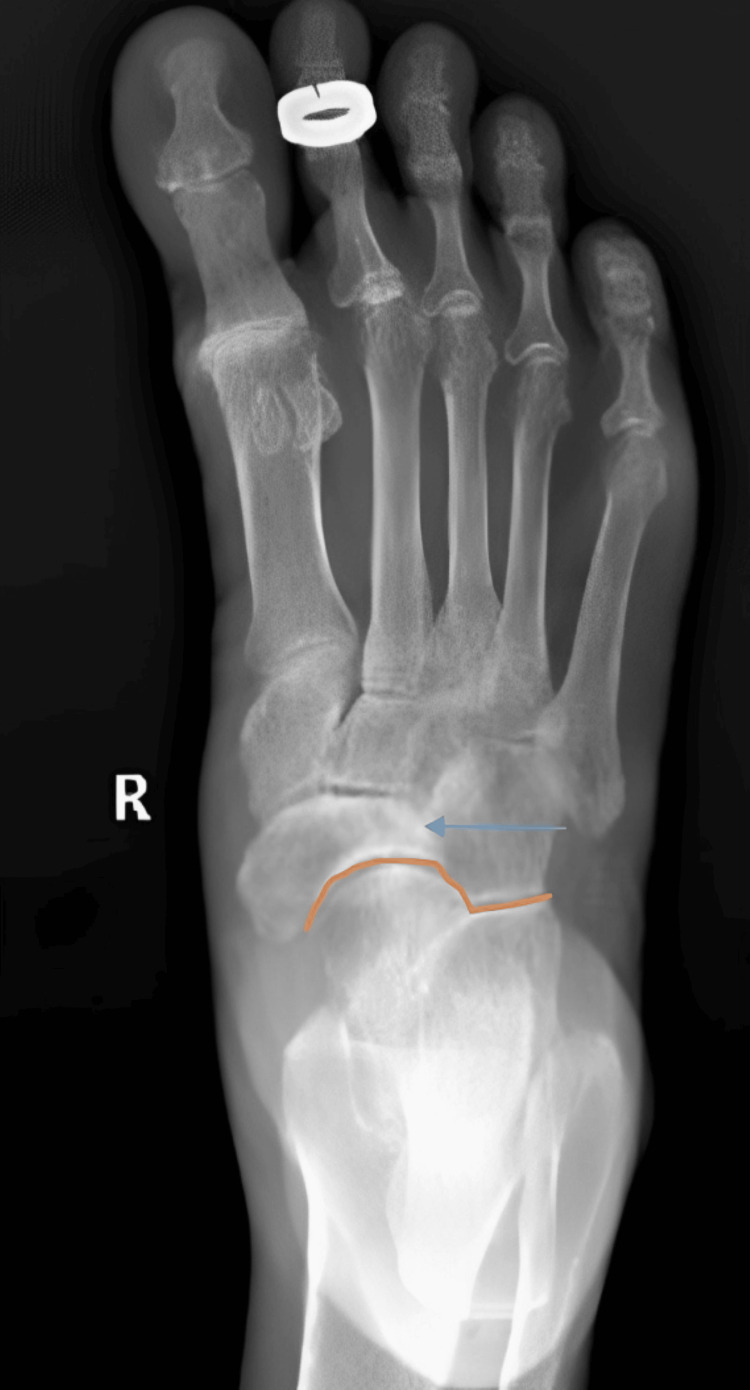
AP radiograph of the right foot showing a partially collapsed and sclerotic lateral half of the navicular bone (blue arrow) and mild lateral subluxation of the talar head with disruption of the cyma line (orange arrow), consistent with Müller-Weiss syndrome

A 69-year-old female presented with pain in the dorsal aspect of the right midfoot and the medial aspect of the ankle. She was initially evaluated with a radiograph, which showed a partially collapsed and sclerotic lateral half of the navicular bone, along with mild lateral subluxation of the talar head and disruption of the cyma line. Based on these findings, a diagnosis of Müller-Weiss syndrome was made (Figure 7).

**Figure 8:**
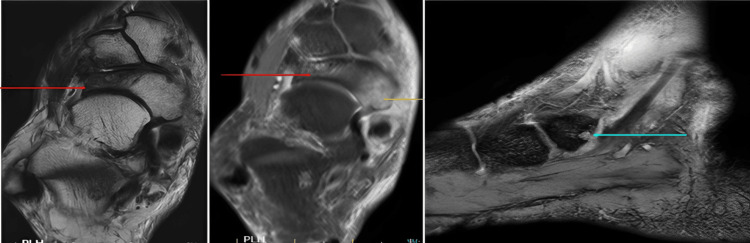
T2, PD-FS axial, and GRE sagittal images (left to right) showing sclerosis with partial collapse and mild STIR hyperintense signal changes involving the lateral half of the navicular bone with no evidence of fragmentation (red arrows) A small erosion with extensive surrounding marrow edema is present in the posteromedial aspect of the navicular bone at the site of insertion of the tibialis posterior tendon (yellow and blue arrows). GRE, gradient-recalled echo; PD-FS, proton density fat-saturated; STIR, short tau inversion recovery

For confirmation of the diagnosis and to evaluate medial-sided ankle pain, an MRI of the right ankle was performed. Imaging showed sclerosis with partial collapse and mild short tau inversion recovery (STIR) hyperintense signal changes involving the lateral half of the navicular bone, with no evidence of fragmentation, and mild secondary degenerative changes in the midfoot joints (talonavicular and naviculocuneiform joints) (Figure 8). A small erosion with extensive surrounding marrow edema was noted in the posteromedial aspect of the navicular bone at the site of insertion of the tibialis posterior tendon (Figure 8).

**Figure 9:**
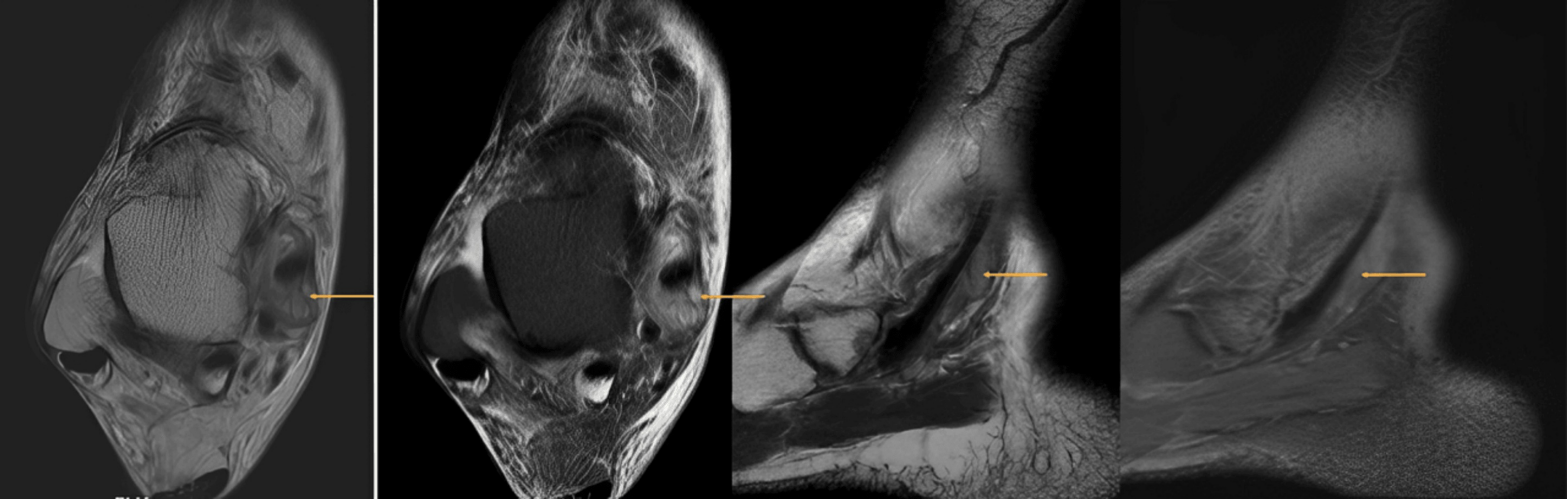
T2 and PD-FS axial and sagittal images showing moderate synovial thickening and effusion along the retro-malleolar tibialis posterior tendon sheath (arrows), suggestive of tenosynovitis PD-FS, proton density fat-saturated

Moderate synovial thickening and effusion were observed along the retro-malleolar tibialis posterior tendon sheath, suggestive of tenosynovitis (Figure 9).

Müller-Weiss syndrome with coexisting inflammatory tibialis posterior tenosynovitis, including bony erosion and marrow edema at its navicular insertion site. Laboratory parameters are summarized in Table 3.

**Table 3:** Laboratory parameters of Case 3 ESR, erythrocyte sedimentation rate; RA factor, rheumatoid factor

Parameter	Value	Normal range
ESR	31 mm/hr	0-20 mm/hr
CRP	0.63 mg/dL	<0.5 mg/dL
RA factor	136.58 IU/ml	<14 IU/ml

The patient is being treated for seropositive erosive RA by the rheumatology team.

## Discussion

The reported cases underscore a compelling, yet often overlooked, intersection between avascular necrosis (ON) and IA. While ON is traditionally associated with well-defined risk factors such as exogenous steroid use, trauma, or alcohol abuse, its co-occurrence with IA, particularly in patients with no prior diagnosis, demands closer examination of the underlying pathomechanisms and the diagnostic utility of advanced imaging.

Pathophysiological link between inflammation and ON

Several mechanistic pathways may explain how systemic inflammation, characteristic of IA, contributes to the development of ON:

Systemic Inflammation and Vascular Dysfunction

The core inflammatory process in diseases such as RA and systemic lupus erythematosus involves the release of pro-inflammatory cytokines, notably tumor necrosis factor-alpha and interleukin-6. These mediators are known to induce endothelial damage and microvascular dysfunction [2]. The resultant vasculitis, or even subclinical endarteritis, can impair circulation to susceptible bone segments, directly causing the ischemic events central to ON [3]. This process is independent of the mechanical effects of joint inflammation.

Hypercoagulable State

Chronic inflammation drives a pro-thrombotic state. The upregulation of tissue factor and suppression of fibrinolysis contribute to hypercoagulability [4]. In the context of the small, end-arterial circulation supplying bones (especially the hip, lunate, and navicular), even minute thrombi can lead to arterial occlusion and subsequent bone infarction [5]. This is particularly relevant in Cases 2 and 3, where seropositivity (suggestive of RA) is strongly associated with an increased risk of thrombotic events.

Local Joint Environment and Pressure Effects (Specific to Epiphyseal ON)

While less relevant for the carpal and tarsal ON observed here, significant synovial inflammation and effusion can acutely elevate intra-articular pressure, compromising intraosseous blood flow, particularly in the femoral head ON [6]. The chronic, erosive nature of RA also locally impacts subchondral bone integrity.

Genetic Susceptibility and Lipid Metabolism

Beyond inflammation, IA may intersect with genetic predispositions for ON. Polymorphisms affecting lipid metabolism (e.g., lipoprotein lipase) or the coagulation cascade (e.g., Factor V Leiden) are known risk factors for idiopathic ON [7]. It is plausible that the added stress of systemic IA triggers ON in genetically predisposed individuals.

Diagnostic significance of advanced imaging

The incidental detection of inflammatory features on MRI in all three patients, initially evaluated solely for suspected ON, highlights the superior sensitivity of MRI in characterizing both bone viability and soft-tissue pathology compared to conventional radiography [8].

Synovitis and Tenosynovitis

The identification of synovial thickening and enhancement (as seen in Cases 1 and 2) or tenosynovitis (as in Case 3) provides direct evidence of an active inflammatory process not detectable on plain films. This finding is a critical diagnostic red flag in the workup of presumed isolated ON.

BME

BME, characterized by T2/STIR hyperintensity, is a non-specific but highly sensitive indicator of an active inflammatory or stress process. While BME is often seen adjacent to necrotic bone, the presence of extensive BME surrounding non-necrotic areas or associated with erosions (as noted in Case 3) strongly suggests active IA or associated mechanical stress [9].

Erosions

The presence of marginal bone erosions in the carpal bones (Cases 1 and 2) or at tendon insertions (Case 3, suggesting enthesitis or erosive tenosynovitis) is pathognomonic for an erosive arthropathy and necessitates immediate rheumatologic referral [10].

## Conclusions

This case series highlights the need for a high index of suspicion for underlying IA when MRI performed for Kienböck’s disease (Cases 1 and 2) or Müller-Weiss syndrome (Case 3) reveals concomitant synovitis, BME, or erosions. The absence of a prior IA diagnosis or history of steroid use underscores the pivotal role of MRI in redefining the diagnosis. Early detection of seropositive erosive arthropathy (as confirmed in Cases 2 and 3) is crucial, as delayed treatment increases the risk of irreversible joint destruction.

The integrated radiological and clinical approach demonstrated here is essential: radiographs identify the classic ON pattern, but MRI provides the critical pathological context that guides systemic management.
